# Is telephone follow-up reliable in maternal and neonatal outcomes surveys in in vitro fertilization?

**DOI:** 10.1186/s12958-022-01001-5

**Published:** 2022-08-23

**Authors:** Ling Sun, Jian Xu, Pei-Ling Liang, Chun-Lin Liu

**Affiliations:** Center for Reproductive Medicine, Guangzhou Women and Children’s Medical Center, Guangzhou Medical University, #9 Jinsui Road, Zhujiang New Town, Tianhe District, Guangzhou City, Guangdong Province 510000 People’s Republic of China

**Keywords:** Reliability, Maternal and neonatal outcome, Follow-up, Telephone, Hospitalized discharge records

## Abstract

**Background:**

Many studies that collect maternal and neonatal outcomes rely on patient self-report phone calls. It is unclear how reliable or accurate these phone call reports are.

**Objective:**

To evaluate the reliability of telephone calls in information collection in IVF.

**Study design:**

The women were interviewed seven days after delivery by a nurse via telephone. The maternal and neonatal outcomes were recorded based on a self-report from one of the spouses. Meanwhile, the standardized electronic hospitalized discharge records were extracted from the hospital medical database. For each case, maternal and neonatal information obtained from telephone interviews and extracted from medical files were compared.

**Results:**

Agreement was classified as “almost perfect, K = 0.81–1.00” for preterm birth, cesarean delivery, low birth weight baby, and macrosomia. The strength of agreement was classified as “moderate, K = 0.41–0.60” for some antepartum complications: gestational diabetes (K = 0.569); pregnancy-induced hypertension (K = 0.588); intrahepatic cholestasis of pregnancy (K = 0.597) and oligohydramnios (K = 0.432). The strength of agreement between telephone interviews and hospitalized discharge records can be classified as “slight (K = 0–0.20)” for some complications: thyroid diseases (K = 0.137), anemia (K = 0.047), postpartum hemorrhage (K = 0.016), and Fetal distress (K = 0.106).

**Conclusion:**

Some variables (preterm birth, cesarean delivery, birth weight) information collected by telephone follow-up were reliable. However, other complications (thyroid diseases, anemia, postpartum hemorrhage, and fetal distress) collected via self-report was non-reliable. Compared with complications during labor, antepartum complications have higher agreement between different follow-up methods. IVF records and hospitalized discharge records should be matched and collected simultaneously when discussing maternal and neonatal outcomes of IVF.

**Supplementary Information:**

The online version contains supplementary material available at 10.1186/s12958-022-01001-5.

## Introduction

Assisted reproductive technology (ART) is a group of medical procedures for treating infertility in which both male and female gametes are handled outside the body to achieve conception [1]. Since its introduction in 1978, ART has contributed to the birth of millions of infants worldwide [2].

With the expanding use of ART, there is a rising concern regarding the safety of these treatments for both mother and child [3]. Hundreds of studies focused on the obstetric and perinatal outcomes of in vitro fertilization (IVF) treatment [4–7]. However, the incidences differed significantly between studies for the same maternal complication (e.g., gestational diabetes). For example, some studies investigated the incidence of gestational diabetes in IVF singleton delivery, the incidence should be comparable theoretically. However, it varied considerably in different studies. A large sample study that enrolled 183,059 IVF single delivery babies reported a 21.1% incidence of gestational diabetes [8], whereas, in other studies, the corresponding rates were 1.4% [9], 5.6% [10] and 12.2% [11], respectively.

The variation in incidence may be due to the heterogeneity of population sampled, but it is more likely due to the inconsistent strategies in data collection. Several methods were described in published literature for obstetric and perinatal information collection, including standardized electronic hospitalized discharge records [8] and national medical birth registries [12]. However, collecting data from medical records derived from large cohorts is time-consuming [13]. For this reason, many studies that collect this information rely on the postal questionnaire or phone calls via patient self-report [14, 15].

The Canadian ART Register collects data on IVF treatment cycles and their outcomes from all ART clinics in Canada. Data related to the pregnancy outcome, birth weight, and congenital malformations are obtained by each clinic through direct follow-up with parents via telephone or mail [16]. Similar methods are applied in the United States; the details of maternal and perinatal complications were collected by nurses who telephoned each patient after delivery and sent to the Society of Assisted Reproductive Technology Outcome Reporting System (SART CORS) [17].

It is unclear how reliable or accurate these questionnaires and phone calls reports are. The data in the SART CORS have been validated annually with some clinics’ medical records. The 2019 ART cycle data validation indicated that most discrepancy rates were low (less than 5%) [18]. The items which were cross-checked in the SART CORS dataset included: patient date of birth, cycle intention, cycle start date, date of egg retrieval, number of eggs or embryos transferred, outcome of ART treatment (i.e., pregnant, or not pregnant), pregnancy outcome (for example, miscarriage, live-birth delivery, or stillbirth), date of pregnancy outcome, number of infants born and patient diagnosis—reason for ART. However, the data on maternal complications is not validated in this chart.

Therefore, it is necessary to conduct a study to evaluate the reliability of the questionnaire and phone calls in maternal and perinatal information collection in IVF treatment. It would be a meaningful addition to the literature, and it would be helpful for subsequent studies in perinatal data collection.

## Materials and methods

### Study design and study participants

This is a cross-sectional study which was conducted in a tertiary maternity hospital between January 2010 and December 2019. In this study, women who were undergoing ART (including in vitro fertilization (IVF), intracytoplasmic sperm injection (ICSI), frozen-thawing embryo transfer (FET)) and with live birth at the same hospital were enrolled in the study. The study was approved by the Independent Ethics Committee of Guangzhou Women and Children’s Hospital (No. 2022-090A01).

According to the routing protocol, the women were interviewed seven days after delivery by a nurse via telephone. The maternal and neonatal outcomes were recorded based on a self-report from one of the spouses. Meanwhile, the standardized electronic hospitalized discharge records were extracted from the hospital medical database. For each case, maternal and neonatal information obtained from telephone interviews and extracted from medical files were compared.

### Data collection via telephone

The couples were informed and signed a follow-up consent form before IVF treatment and were interviewed by telephone seven days after delivery. Data collected via telephone included: date of delivery, mode of delivery, number of children born, gender and birth weight of each baby, congenital malformations of each baby, and maternal/neonatal complications. Data collected via telephone were recorded in the ART database.

To ensure the consistency of the follow-up process, all of the nurses were trained, and a uniform follow-up questionnaire was applied (Additional file 1). If the couples did not answer the first call, additional calls were made three or four days later to maximize the follow-up rate.

When extracting variables from the ART database, personal identification number of the women, IVF/ICSI, fresh/cryopreserved, date of embryo transfer, and number of embryos transferred were extracted for further analysis.

### Data collection from standardized electronic hospitalized discharge records

In standardized electronic hospitalized discharge records, all diagnoses of disorders and diseases were coded using the *International Statistical Classification of Diseases and Related Health Problems, Eleventh Revision* (*ICD-11*) [19]; all operating procedures were coded by using the *ICD-11* and the *International Classification of Diseases, 9th Revision. Clinical Modification* (*ICD-9-CM*) [20].

Before the linkage process, a limited data file was generated from standardized electronic hospitalized discharge records, containing only the following factors: women’s personal identification number, woman’s first, middle name or initial, and last names, date of hospitalized discharge, and whole items of discharge diagnosis.

### Linkage procedure

We linked the ART database and hospitalized discharge records. In the first step, the women’s personal identification number was cross-linked between the two databases to ensure proper identity recognition. Then the date of hospitalized discharge was linked to the date of embryo transfer to exclude the delivery followed by a spontaneous conception of the same woman. Thirdly, duplicated records were excluded if the women were hospitalized several times during the same conception. Fourthly, the study population was limited to delivery births only; hospitalized discharge without delivery record is also excluded.

### Definition of maternal complications neonatal outcomes

Maternal chronic diseases were defined as chronic diseases the pregnant woman had before pregnancy, including thyroid diseases, anemia, and other diseases. Maternal complications were defined as disorders that developed during pregnancy, including pregnancy-induced hypertension (persistent blood pressure ≥ 140/90 mmHg was recorded after 20 weeks of gestation in a previously normotensive woman, preeclampsia and eclampsia), gestational diabetes mellitus, placenta previa, placental abruption, oligohydramnios, polyhydramnios, preterm birth (gestational age at birth, 28–36 weeks), cesarean delivery, abnormal placental cord insertion, postpartum hemorrhage (bleeding volume ≥ 500 mL after vaginal delivery or ≥ 1000 mL after cesarean delivery), and intrahepatic cholestasis of pregnancy. Neonatal outcomes were defined as neonatal complications that developed before or after birth until discharge, including fetal distress, low birth weight (birth weight < 2500 g), macrosomia (birth weight > 4000 g).

Among these variables, low birth weight and macrosomia were identified according to birth weights reported in the records. Preterm birth was accounted for according to birth date and embryo transfer date. Other variables were identified according to the related *ICD-9-CM* or *ICD-10* codes.

### Statistical analysis

Categorical variables were expressed as frequency and percentage. Cohen’s kappa (κ) statistics were used to investigate the agreement between records from telephone follow-up and of hospitalized discharge records. Kappa coefficients were interpreted as follows: almost perfect (0.81–1.00), substantial (0.61–0.80), moderate (0.41–0.60), fair (0.21–0.40), and slight (0–0.20) [21, 22]. All data analyses were performed using SPSS for windows 23.0. (IBM, Armonk, NY). *P* values < 0.05 were considered significant.

## Results

### Study population

**Fig. 1:**
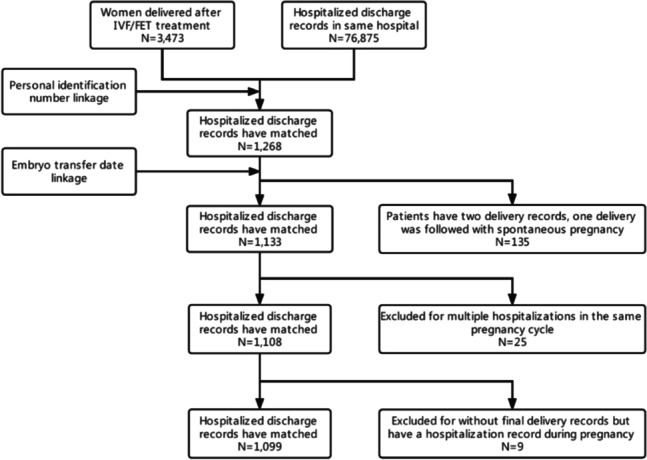
Flowchart of data according to eligibility for inclusion in this study

Totally 3,473 women who were pregnant after IVF/ICSI/FET treatment in Guangzhou women and children’s hospital were enrolled for cross-link, and 1,268 hospitalized discharge records were matched. Then 135 deliveries records in hospitalized discharge records were excluded for there were no corresponding embryo transfer records, and the deliveries were considered to follow with spontaneous pregnancies. Twenty-five women were hospitalized more than one time during pregnancy for some reasons, then duplicated medical records were excluded. Nine medical records were excluded, for there was a record of hospitalization during pregnancy but no final delivery records. Finally, 1,099 records were included in the study. The detailed linkage results are shown in Fig. 1.

Of the 1,099 delivery records, 771 (70.2%) were singleton deliveries, and 328 (29.8%) were twin deliveries. Finally, a total of 1099 women and 1,427 newborns were enrolled in the study.

### Maternal and neonatal outcomes

More than ten types of maternal and neonatal complications were recorded in a phone interview, whereas a much greater variety of complications were recorded in hospitalized discharge records files.

Table 1 illustrates agreement between self-report by telephone and medical records for binary variables. The agreement was classified as “almost perfect, κ = 1.00” for the following variables: preterm birth, cesarean delivery (97.6% in twin pregnancy, 49.5% in single delivery), low birth weight baby, and macrosomia.

**Table 1:** Agreement between self-report by telephone and medical records for maternal and neonatal outcomes

	Follow-up by telephone *N* = 1099	Follow-up by obstetric files *N* = 1099	Κ^a^	*p*
Maternal chronic diseases				
Thyroid diseases	0.4% (4/1099)	4.7% (52/1099)	0.137	0.000
Anemia	0.5% (5/1099)	15.3% (168/1099)	0.047	0.000
Maternal outcomes				
Gestational diabetes mellitus	12.7% (140/1099)	26.8% (295/1099)	0.569	0.000
Pregnancy-induced hypertension	4.7% (52/1099)	10.6% (117/1009)	0.588	0.000
Intrahepatic cholestasis of pregnancy	0.5% (6/1099)	1.3% (14/1099)	0.597	0.008
Placenta previa	1.0% (11/1099)	4.1% (45/1099)	0.383	0.000
Placental abruption	0.2% (2/1099)	1.4% (15/1099)	0.233	0.000
Oligohydramnios	0.6% (7/1099)	2.3% (25/1099)	0.432	0.000
Polyhydramnios	0.3% (3/1099)	2.4% (26/1099)	0.203	0.000
Preterm birth	16.1% (177/1099)	16.1% (177/1099)	1.000	1.000
Cesarean section	63.9% (702/1099)	63.9% (702/1099)	1.000	1.000
Abnormal placental cord insertion	0.8% (9/1099)	4.2% (46/1099)	0.318	0.000
Postpartum hemorrhage	(1/1099)	10.2 (112/1099)	0.016	0.000
neonatal outcomes				
Fetal distress	0.1% (2/1427)	2.5% (35/1427)	0.106	0.000
Low birth weight	25.8% (368/1427)	25.8% (368/1427)	1.000	1.000
Macrosomia	2.2% (31/1427)	2.2% (31/1427)	1.000	1.000

^a^Kappa coefficients: almost perfect (0.81–1.00), substantial (0.61–0.80), moderate (0.41–0.60), fair (0.21–0.40), and slight (0–0.20)

Compared with the phone interview, hospitalized discharge records file reported a higher proportion of other complications. The strength of agreement was classified as “moderate, κ = 0.41–0.60” for the following variables: gestational diabetes (κ = 0.569); pregnancy-induced hypertension (κ = 0.588); intrahepatic cholestasis of pregnancy (κ = 0.597) and oligohydramnios (κ = 0.432). It is of note that gestational diabetes mellitus and pregnancy-induced hypertension have higher self-report rates. However, compared with hospitalized discharge records, gestational diabetes mellitus was not frequently recorded in the telephone interview, with the information missing in 155 of 295 sets of notes. The total incidence of gestational diabetes mellitus was 12.7% in a telephone interview; it increased to 26.8% in hospitalized discharge records. Similar results were recorded in pregnancy-induced hypertension (4.7% vs. 10.6%). Detailed data are shown in Table 1.

Agreement for some complications were classified as “fair, κ = 0.21–0.40”, including placenta previa (κ = 0.383), placental abruption (κ = 0.233), polyhydramnios (κ = 0.233) and Abnormal placental cord insertion (κ = 0.318). The strength of agreement between telephone interviews and hospitalized discharge records can be classified as “slight (κ = 0–0.20)” for the remainder of complications: thyroid diseases (κ = 0.137), anemia (κ = 0.047), postpartum hemorrhage (κ = 0.016), and Fetal distress (κ = 0.106). Detailed data are shown in Table 1.

## Discussion

This is the first study to assess the consistency of telephone follow-up and hospitalized discharge records in maternal and neonatal complication collection. We found that the information on preterm birth, cesarean delivery, low birth weight baby, and macrosomia were in complete agreement between the two methods. Other maternal and neonatal complications were rarely reported in telephone interviews, much lower than hospitalized discharge records.

Four variables, including cesarean delivery, preterm birth, low birth weight baby, and macrosomia, were in complete agreement. This finding is in line with previous study, which have reported correlations of around 0.989 in birth weight [13]. However, this study assessed consistency for one variable merely, the birth weight. The reason why these four variables are perfectly consistent may be due to several factors: firstly, cesarean section was an operation that the patient will remember very well; secondly, we asked the couples about the birth date and birth weight of the newborn in detail in telephone follow-up, then we calculated preterm birth, low birth weight baby and macrosomia according to the time of embryo transfer and the criterion of low birth weight. Parents’ recall of infant birth weight and birthday was highly accurate compared to hospitalized discharge records, making the correlation between the two sources of information close to unity.

Except for the four obstetric outcomes mentioned above, the incidence of maternal and neonatal outcomes was significantly higher in obstetric discharge records than in telephone follow-up records. This means a high percentage of complications were missing reported by telephone follow-up.

Agreement between self-report and obstetric discharge records was classified as “moderate” or “fair” for antepartum complications included in this study. In comparison, it was classified as “slight” for complications during labor.

The relatively high self-reported rate for antepartum complications may be due to these complications onset at the second or the third trimester of pregnancy; therefore, the women were informed many times during prenatal examination; and due to the harmfulness of gestational diabetes mellitus and pregnancy-induced hypertension, the women pay more attention to monitoring them. However, obstetric complications during labor, such as postpartum hemorrhage and placental abnormalities, are most likely missed in self-reported. This may be due to patients may pay more attention to the neonatal health of IVF babies while ignoring those less severe obstetric complications.

An important strength of the study is that all cases have been follow-up by the same standard procedures and equally trained professionals. On the contrary, as results were based on single-center, results should be considered with caution, due to the efficiency of telephone follow-up may vary from center.

In conclusion, some variables (preterm birth, cesarean delivery, birth weight) information collected by telephone follow-up were reliable. However, other complications (thyroid diseases, anemia, postpartum hemorrhage, and fetal distress) collected by self-reported via telephone were non-reliable. Compared with complications during labor, antepartum complications have higher agreement between different follow-up methods. IVF records and hospitalized discharge records should be matched and collected simultaneously when discussing maternal and neonatal outcomes of IVF.

## Supplementary Information

**Additional file 1:** **Supplementary Table 1.** Questionnaire of follow-up by telephone review.

## Data Availability

The data is not publicly shared and please contact author for data requests.
