# Supplementary material for: Is telephone follow-up reliable in maternal and neonatal outcomes surveys in in vitro fertilization?
Source: Reprod Biol Endocrinol. 2022 Aug 23;20:128. doi: 10.1186/s12958-022-01001-5 (PMC9396850; doi:10.1186/s12958-022-01001-5)
Supplement: Supplementary file 1 — Additional file 1: Supplementary Table 1. Questionnaire of follow-up by telephone review. [file 12958_2022_1001_MOESM1_ESM.docx]

Supplementary table 1: Questionnaire of follow-up by telephone review

| Date of delivery | 1. Date of delivery: |
| --- | --- |
| Delivery mode | 1. Delivery mode   □Natural labor, □Forceps Delivery, □Cesarean section   1. If the delivery mode was selected except "natural labor," please complete the following question.   Reasons: |
| Maternal complications | 1. Maternal complications: □yes, □no; If "Yes" was answered, please complete the following question.   □pregnancy-induced hypertension, □gestational diabetes, □postpartum hemorrhage, □abnormal amniotic fluid, □abnormal of the placenta,  □others |
| Neonatal information | 1. The number of the babies delivered:   □Single baby; □Twin babies   1. Gender and weight of the babies:   baby 1: □Male, □Female; birth weight g;  baby 2: □Male, □Female; birth weight g;   1. Neonatal complications: □yes, □no; If "Yes" was answered, please complete the following question.   □Fetal distress, □others |
